# Speckleplethysmographic (SPG) Estimation of Heart Rate Variability During an Orthostatic Challenge

**DOI:** 10.1038/s41598-019-50526-0

**Published:** 2019-10-01

**Authors:** Cody E. Dunn, Derek C. Monroe, Christian Crouzet, James W. Hicks, Bernard Choi

**Affiliations:** 1 Beckman Laser Institute and Medical Clinic, University of California, Irvine, California 92612 USA; 2 Department of Biomedical Engineering, University of California, Irvine, California 92697 USA; 3 Edwards Lifesciences Center for Advanced Cardiovascular Technology, University of California, Irvine, California 92697 USA; 4 Department of Neurology, University of California, Irvine, California 92697 USA; 5 Department of Ecology and Evolutionary Biology, University of California, Irvine, California 92697 USA; 6 Department of Surgery, University of California, Irvine, California 92697 USA

**Keywords:** Diagnosis, Translational research

## Abstract

Heart rate variability (HRV) provides insight into cardiovascular health and autonomic function. Electrocardiography (ECG) provides gold standard HRV measurements but is inconvenient for continuous acquisition when monitored from the extremities. Optical techniques such as photoplethysmography (PPG), often found in health and wellness trackers for heart rate measurements, have been used to estimate HRV peripherally but decline in accuracy during increased physical stress. Speckleplethysmography (SPG) is a recently introduced optical technique that provides benefits over PPG, such as increased signal amplitude and reduced susceptibility to temperature-induced vasoconstriction. In this research, we compare SPG and PPG to ECG for estimation of HRV during an orthostatic challenge performed by 17 subjects. We find that SPG estimations of HRV are highly correlated to ECG HRV for both time and frequency domain parameters and provide increased accuracy over PPG estimations of HRV. The results suggest SPG measurements are a viable alternative for HRV estimation when ECG measurements are impractical.

## Introduction

The autonomic nervous system (ANS) acts unconsciously to control various organ functions, including the rhythmicity and contractility of the heart. The interplay between the sympathetic and parasympathetic branches of the ANS result in heart rate variability (HRV). A reduction in HRV reflects an increase in sympathetic input and withdrawal of parasympathetic input to the heart. HRV, a class of metrics derived from variability in R-R intervals typically measured using electrocardiography (ECG), has implications for cardiovascular and neurological health^1,2^. Furthermore, HRV can be used to prescribe exercise in young adults^3^, as a biomarker of overtraining in athletes^4–6^, and as a signal to guide biofeedback training designed to reduce stress and anxiety^7^. Accurate remote monitoring of HRV (i.e., at home, field-side, or by athletic trainers) is necessary to mitigate the negative effects related to competitive athletic performance and inform rest requirements^6^.

Currently, ECG measurements provide gold-standard HRV monitoring. ECG acquired from the chest requires thoracic electrodes, and these electrodes are highly susceptible to noise from poor contact and motion artifact. Unfortunately, routine, remote HRV monitoring is limited due to convenience, comfort, and loss of accuracy^8^. Furthermore, traditional ECG measurements utilize adhesive for the electrodes that can be uncomfortable during removal when compared to an optical finger clip device^9^. Various groups have attempted to address the limitations of ECG monitored HRV by estimating HRV with cheaper photoplethysmography (PPG) technology (Note: PPG estimates of HRV may be referred to as pulse rate variability (PRV) in some of the literature, but we elected to maintain the PPG HRV nomenclature for simplicity and consistency. PPG HRV is equivalent to PPG PRV in this work)^8,10,11^.

**Figure 1:**
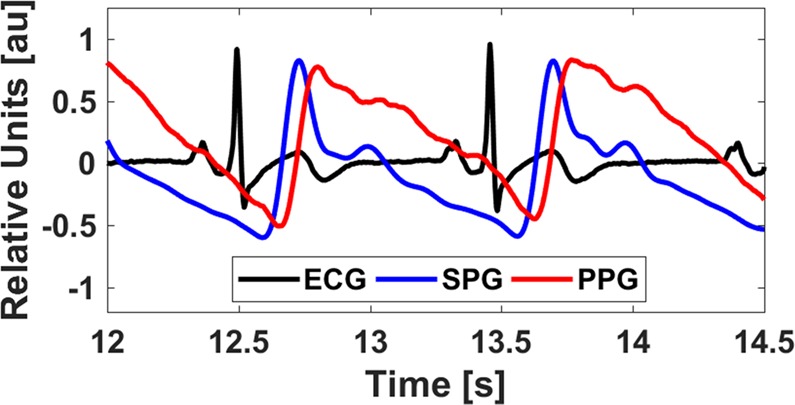
An *in vivo* comparison of ECG (black), SPG (blue), and PPG (red) waveforms measured from a single subject. Because of the pulse transit time from the chest (ECG) to the finger (SPG/PPG), the ECG has its R peak before the SPG peak. SPG peaks before PPG.

Transmittance PPG, the signal used in pulse oximetry, measures changes in intensity due to light absorption caused by the dilation and constriction of arteries and arterioles in the finger due to pulsatile blood flow. Nonetheless, HRV approximated from optical finger measurements loses accuracy due to significant peak time delays (Fig. 1) related to various factors such as arterial stiffness, vascular tone, and height^12^. PPG HRV has proven accurate only for healthy subjects at rest, but loses accuracy with increasing physical stress due to motion artifact and the noted time delay^11^. The differences are more easily noticeable in the frequency domain, especially in the high frequency band (0.15–0.4 Hz)^13^ due to pulse transit time (PTT) variability and respiratory activity^12^. Novel phone-based reflectance PPG measurements provide improved accessibility but face the same inherent limitations as transmittance PPG as well as reduced signal quality^14,15^. Other state-of-the-art methods used for approximation of HRV lack feasibility outside of a lab-based setting^16^.

Speckleplethysmography (SPG), an optical signal that measures changes in blood flow using laser speckle imaging^17^, provides an improved signal-to-noise ratio^18^ and robustness in the presence of motion artifact and cold temperatures as compared to PPG^19^ (Fig. 1). Similar to PPG, it can be measured from the finger and processed in real-time^19^. In addition, SPG peaks before PPG, which should improve accuracy and reduce the impact of vascular compliance on HRV estimation (Fig. 1). The components required for SPG acquisition, a budget camera and laser pointer, are relatively inexpensive^20^. To date, SPG has not been reported on in the literature as a measure of HRV. Given the aforementioned benefits of SPG, we determined the accuracy of SPG during an orthostatic challenge for estimations of HRV.

## Results

### Artifact correction

**Figure 2:**
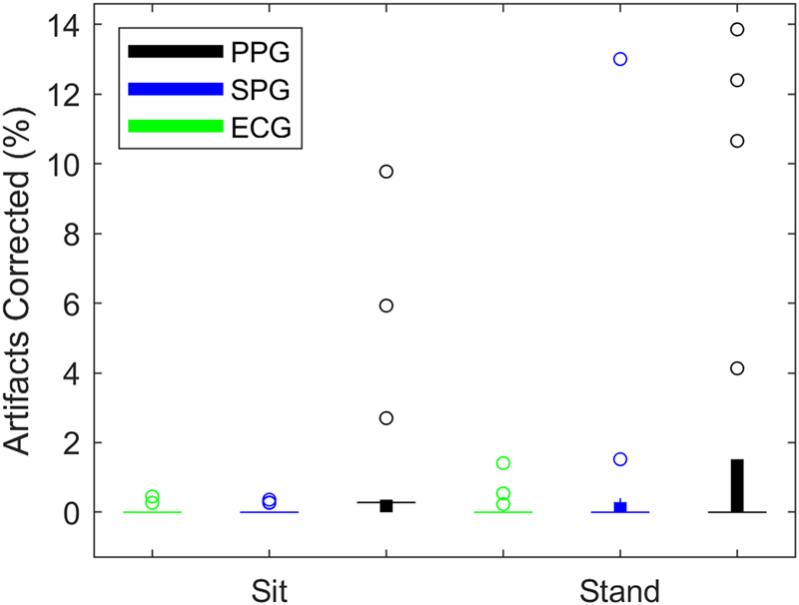
A box plot comparison of the percentage of artifacts corrected for ECG (green), SPG (blue), and PPG (black) during the orthostatic challenge (n = 17). The circles represent outliers. Motion artifact and noise negatively impacted the peak detection algorithm accuracy, especially for PPG.

Although the subjects were asked to remain still, the impact of motion artifact on signal quality varied for each subject. Furthermore, the peak detection algorithm had higher accuracy for some subjects depending on noise inherent to the signals based on factors such as individual blood flow^21^ and skin tone^22^. We noted the percentage of corrected artifacts in Kubios for each time series (Table 1; Fig. 2). As expected, the standing data had a larger percentage of artifacts. The seated PPG signals (black) had a larger number of corrected artifacts than the seated ECG (green) and SPG (blue) signals.

**Table 1:** Corrected Artifacts (n = 17).

	ECG Sit	SPG Sit	PPG Sit	ECG Stand	SPG Stand	PPG Stand
Minimum (%)	0	0	0	0	0	0
Median (%)	0	0	0.28	0	0	0
Maximum (%)	0.45	0.36	9.78	1.41	13.01	13.86

A comparison of the minimum, median, and maximum percentage of corrected artifacts for each measurement technique during the orthostatic challenge (n = 17).

### Time domain parameters

We independently compared the SPG and PPG estimates of HRV to the ECG HRV during both the sitting and standing periods of the orthostatic challenge. We plotted the standard deviation of normal to normal R-R intervals (SDNN; ms) from the 17 subjects, which reflects both the short-term and long-term cyclic components responsible for variability during the recording period^13^, given by the equation^23^:

1 $$SDNN=\sqrt{\frac{1}{N-1}\mathop{\sum }\limits_{n-1}^{N}{(R{R}_{n}-\overline{RR})}^{2}}.$$

In addition, we plotted the root mean square of the successive differences (RMSSD; ms), which reflects vagal tone^24^ and short-term variability, from the 17 subjects, given by the equation^23^:

2 $$RMSSD=\sqrt{\frac{1}{N-1}\mathop{\sum }\limits_{n=1}^{N-1}{(R{R}_{n+1}-R{R}_{n})}^{2}.}$$

**Figure 3:**
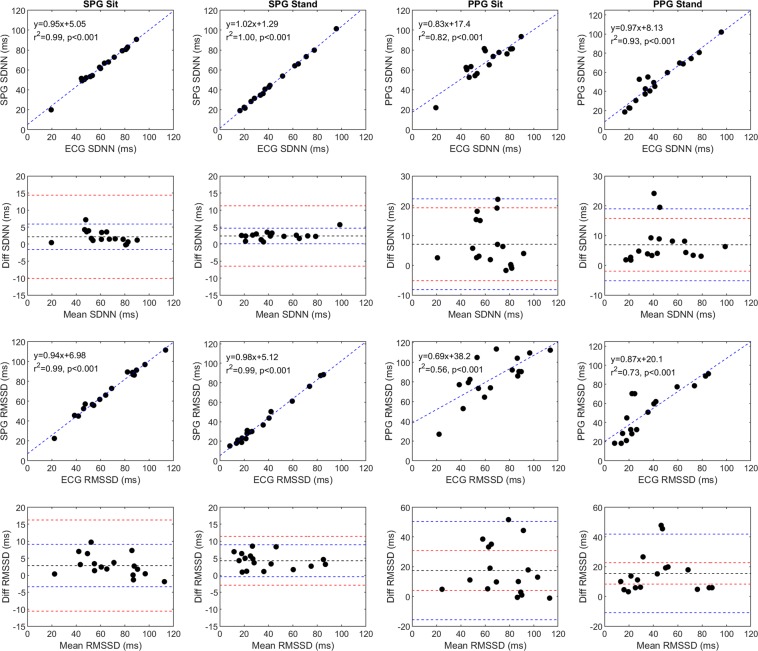
A comparison of SPG, PPG, and ECG HRV time domain parameters (SDNN; ms and RMSSD; ms) for the 17 subjects during both sitting and standing conditions. SPG and PPG are compared to ECG on scatter plots with a line of best fit and the line of best fit equation, the Pearson’s correlation coefficient, and significance in the upper left corner of the plot. Each Bland-Altman plot corresponds to the scatter plot directly above it, with the mean difference (black line), the 95% upper and lower limits of agreement (blue dashed lines), and the acceptable upper and lower limits of agreement (red dashed lines) also plotted. It should be noted that the Bland-Altman plots for SPG and PPG have different y-axis scales.

We also generated the corresponding Bland-Altman plots (Fig. 3). The SPG results are highly correlated to the ECG results and fall within the predetermined acceptable limits of agreement for both the sitting (Fig. 3, column 1) and standing (Fig. 3, column 2) periods. The PPG results are less correlated to the ECG results (Fig. 3, columns 3 and 4) than the SPG results and some of the data points fall outside the acceptable limits of agreement. Interestingly, the PPG results improved when the subjects went from sitting to standing.

### Frequency domain parameters

**Figure 4:**
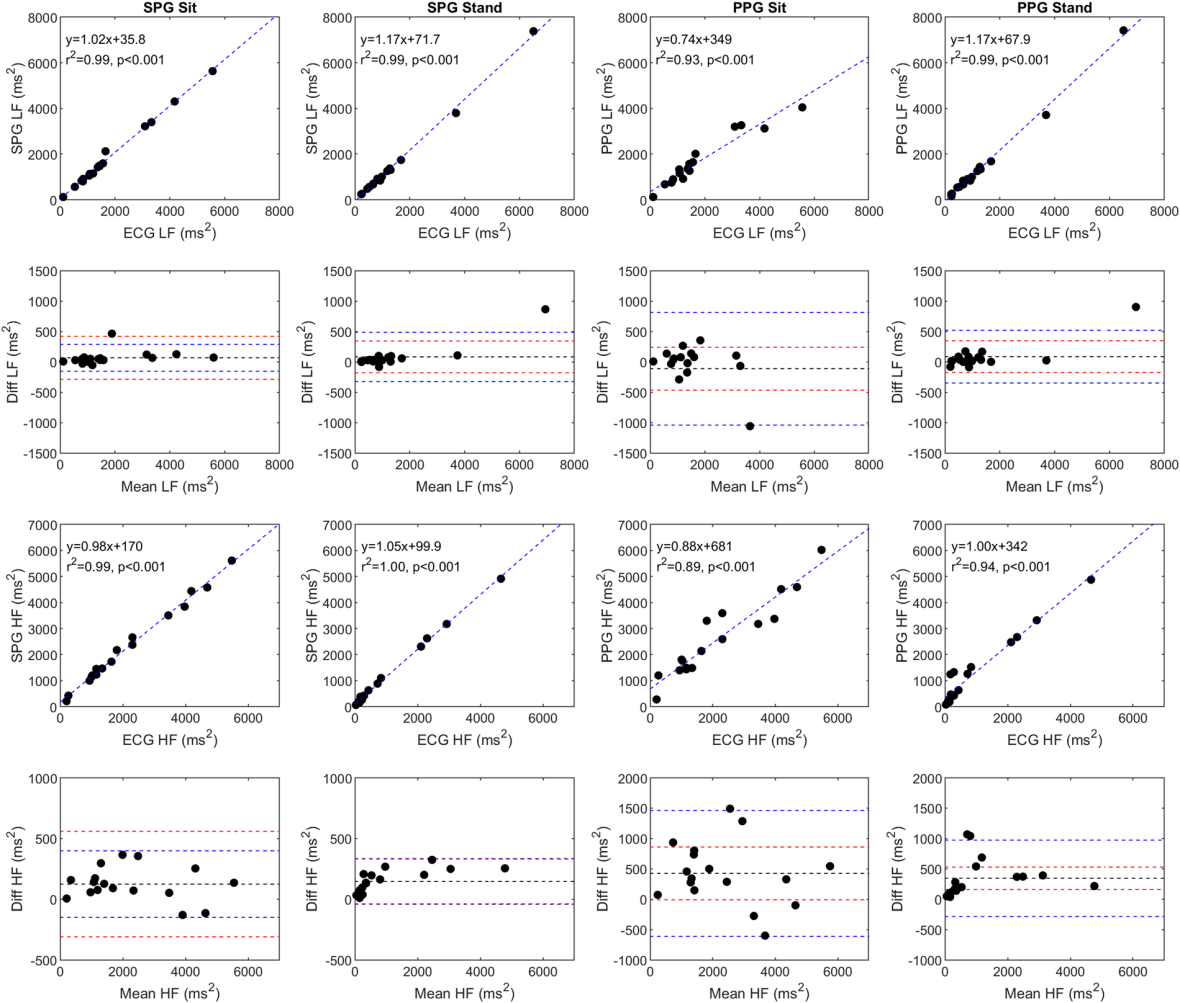
A comparison of SPG, PPG, and ECG HRV frequency domain parameters (LF; ms^2^ and HF; ms^2^) for the 17 subjects during both sitting and standing conditions. SPG and PPG are compared to ECG on scatter plots with a line of best fit and the line of best fit equation, the Pearson’s correlation coefficient, and significance in the upper left corner of the plot. Each Bland-Altman plot corresponds to the scatter plot directly above it, with the mean difference (black line), the 95% upper and lower limits of agreement (blue dashed lines), and the acceptable upper and lower limits of agreement (red dashed lines) also plotted. It should be noted that some of the Bland-Altman plots for SPG and PPG have different y-axis scales.

In addition, we compared the low frequency (LF; ms^2^) and high frequency (HF; ms^2^) components of the three signals, which span the 0.04 Hz–0.15 Hz and 0.15 Hz–0.4 Hz bands, respectively^24^ (Fig. 4).The low frequency band originates from long-term regulation mechanisms such as thermoregulation and hormonal mechanisms, while the high frequency band originates from vagal tone and relates to the respiratory cycle^24^. Once again, the SPG results are highly correlated to the ECG results during both standing and sitting. However, for the SPG versus ECG Bland-Altman LF plots from both the sitting and standing measurements, there is a single point that falls outside of the acceptable limits of agreement. All HF SPG measurements fall within the acceptable limits of agreement. Similar to the time domain measurements, the PPG measurements have a larger correlation to the ECG results when the subjects are standing. All of the PPG Bland-Altman plots have one or more points that fall outside the acceptable limits of agreement.

**Figure 5:**
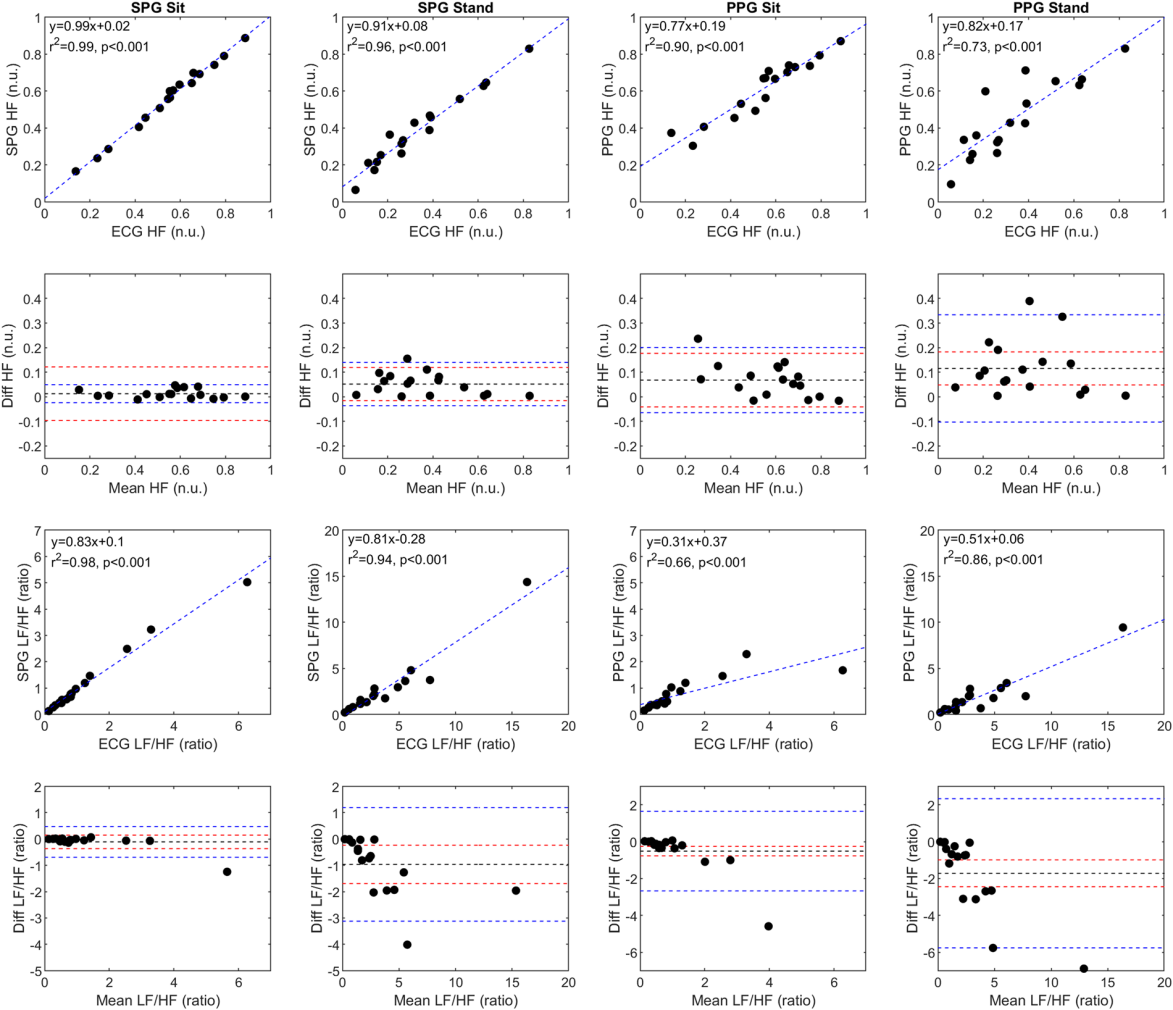
A comparison of additional SPG, PPG, and ECG HRV frequency domain parameters (normalized HF; normalized units (n.u.) and LF/HF; ratio) for the 17 subjects during both sitting and standing conditions. SPG and PPG are compared to ECG on scatter plots with a line of best fit and the line of best fit equation, the Pearson’s correlation coefficient, and significance in the upper left corner of the plot. Each Bland-Altman plot corresponds to the scatter plot directly above it, with the mean difference (black line), the 95% upper and lower limits of agreement (blue dashed lines), and the acceptable upper and lower limits of agreement (red dashed lines) also plotted.

The last set of HRV parameters we compared combine the HF and LF components from above^25^. First, we examined the normalized HF (normalized units or n.u.), HF (ms^2^)/[HF (ms^2^) + LF (ms^2^)]. Both the sitting and standing SPG measurements correlated well with the ECG measurements and better than the PPG measurements (Fig. 5, rows 1 and 2). The seated SPG measurements fell within the acceptable limits of agreement, but one point from the standing SPG measurements fell outside the acceptable limits of agreement. Next, we compared the LF/HF ratio, which represents a mix of sympathetic and vagal activity^24^, between the three signals (Fig. 5, rows 3 and 4). Based on the Bland-Altman plots, SPG and PPG appear to underestimate the LF/HF ratio at higher ratios. SPG has a higher correlation with ECG as compared to PPG with ECG during both the sitting and standing conditions.

## Discussion

HRV measurements provide rich information concerning autonomic regulatory capacity as an indicator of cardiovascular and neurological function. However, the gold standard technique for the determination of HRV parameters involves ECG, which is limited in situations where peripheral measurements are desirable due to convenience and motion artifact^8^. Substitute techniques that provide estimates of HRV parameters (i.e. PPG) address these limitations at the cost of accuracy^26^. Our results demonstrate that SPG estimates both time and frequency domain parameters of HRV with relatively high accuracy during both sitting and standing conditions, suggesting SPG could prove beneficial for remote measurements of ANS function. When examining the Bland-Altman plots, all SPG estimations remain within the acceptable limits of agreement for time domain measurements. Accuracy decreases for frequency domain measurements. The correlation coefficients decrease for the HF (n.u.) and LF/HF ratio because of compounding errors. Our data suggest that SPG is more accurate than PPG across all HRV parameters assessed in this study when collected from the same device. As expected, a larger percentage of artifacts required correction during standing than sitting. One possible explanation for the improved PPG correlations during standing is that the signal quality improved such that the peaks were sharper, which allowed for improved peak detection when corrections were unnecessary^11^. The improved signal quality may be attributed to the increased blood volume in the finger due to gravity and location of the finger relative to the heart while standing^27^.

We acknowledge that the study does have some limitations. First, we did not control the room temperature, although it remained relatively constant for each subject measurement and did not shift more than 4 °C between subjects. Colder temperatures reduce the signal quality of PPG, which in turn makes accurate peak detection difficult^28^. Furthermore, we did not apply more complex signal processing methods for filtering of the signals prior to peak detection^29,30^. We deemed using SPG peaks to assist with PPG peak detection reasonable because both signals are collected by the same imaging device. With preliminary analysis, we observed that PPG peak detection results using the same filtering techniques applied to the SPG signal (described in the Methods section) were poor, possibly because the PPG signal has a smaller signal to noise ratio than SPG^18^. To aid in future motion artifact identification and correction, we suggest collecting accelerometer data during an orthostatic challenge^31^. The subjects involved in this study were aerobically trained athletes that may have a reduced minimum rise time^32^, which would improve HRV estimation accuracy; we acknowledge that the results of this study may not extend to the general population. PPG estimations of HRV become less accurate as subjects age due to increased arterial stiffness and more PTT variability, but we predict SPG would be more robust to these changes based on past results^19^.

SPG faces many of the same limitations as PPG because of the PTT separating thoracic electrical measurements at the heart from optical measurements at the fingertip. On the other hand, the reduced susceptibility to motion artifact and temperature during both sitting and standing conditions suggest SPG measurements are preferable to PPG measurements for estimating HRV. To the authors’ knowledge, this is the first study to directly compare SPG and ECG as a substitute measurement for HRV, and the correlation coefficients obtained support the notion that SPG HRV estimations are preferable to PPG HRV estimations in settings when ECG HRV cannot be collected. SPG estimations of HRV can aid in the prevention of over-training by enabling remote and convenient monitoring of decreases in HRV. Furthermore, recent studies noticed a decrease in HRV after concussions^6,33,34^. SPG could provide a method for on-field monitoring of head impacts.

## Materials and Methods

### Subject recruitment

We recruited 17 healthy intercollegiate athletes (9 males, 23 ± 3.74 years; 8 females, 19.25 ± 1.28 years) who were undergoing preseason ECG measurements as part of a study designed to monitor athletes for head impact exposure. The subjects were instructed to avoid caffeine consumption for six hours prior to the measurement. All measurements were done in accordance with human subject protocols approved by the Institutional Review Board at University of California, Irvine (HS#2008-6307 and HS#2014-1338). Informed consent was obtained from all subjects.

### Equipment

We utilized a commercial finger-clip blood-flow sensing device (Flowmet, Laser Associated Sciences (LAS), Inc., Irvine, CA) connected to a Microsoft Surface Pro 5 with LAS software for simultaneous SPG and PPG signal acquisition. A coherent light source (785 nm) transilluminated each subject’s finger and a 752-pixel × 480-pixel CMOS array detected the transmitted light, similar to a pulse oximeter. The Flowmet acquired images at 250 Hz and the exposure time was adjusted to ensure adequate signal given different finger thicknesses and skin tones. For wireless ECG acquisition at 2000 Hz, we used a Nomadix Wireless Receiver with ECG Amplifier (BIOPAC Systems, Inc., Goleta, CA). We designed and built a circuit for optical triggering of the Flowmet and electrical triggering of the BIOPAC system to ensure temporal synchronization of the two monitoring devices.

### Data collection

**Figure 6:**
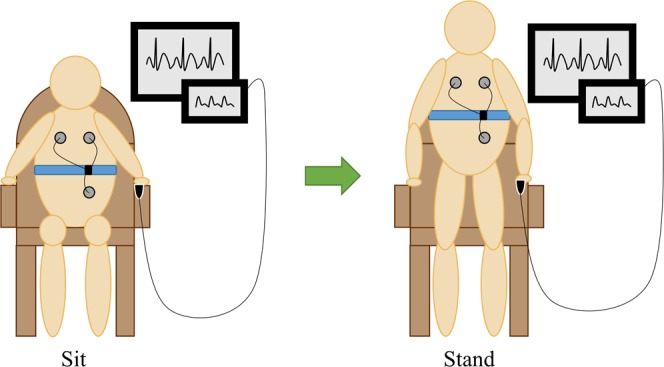
Subjects remained seated for 5 minutes while a finger-clip device simultaneously collected SPG and PPG data and a wireless system collected ECG data. After a 30 second transition period for the subject to go from sitting to standing, 5 minutes of SPG, PPG, and ECG data were collected while the subject remained standing.

We placed the Flowmet on the left index finger and the wireless ECG system in the lead II configuration on the chest of the subject. Next, we instructed the subject to remain seated still during the measurement with the room lights off and then triggered the data acquisition protocol for both devices. The room temperature ranged from 20 °C to 24 °C. We continuously collected SPG, PPG, and ECG data with the subject first seated for 5 minutes and then standing for 5 minutes (Fig. 6).

### Data analysis

We processed the raw ECG data using MATLAB software (R2018b, Mathworks, Inc., Natick, MA) for peak detection of the R wave and subsequent R-R interval calculation. The R-R intervals for seated and standing measurements were separated and saved as two text files for analysis in Kubios HRV Standard 3.1.0 (Kubios, Kuopio, Finland).

The Flowmet outputs raw data as average intensity, *I*, in camera counts from 0–255 and average speckle contrast squared, *K* ^2^, from 0–1 for each image. We converted the average intensity to PPG according to the Beer-Lambert Law^35^:

3 $$PPG=\frac{1}{\mathrm{ln}(I)}$$

where *PPG* is measured in arbitrary units. Speckle contrast was converted to SPG, which correlates linearly with blood flow^36^, using the simplified speckle imaging equation^37^:

4 $$SPG=\frac{1}{2T{K}^{2}}$$

where *SPG* is measured in arbitrary units and *T* is the exposure time of the Flowmet image detector.

**Figure 7:**
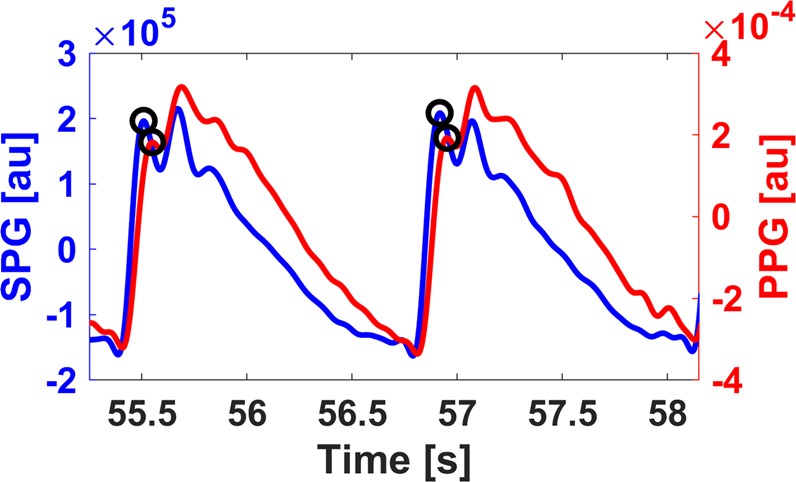
SPG (blue) and PPG (red) data taken from a subject while seated. For consistency in SPG peak detection, the first peak after a major trough for each period was used for SPG N-N interval analysis. The first PPG peak after the nearest detected SPG peak was used for PPG N-N interval analysis.

To process the raw Flowmet data, we wrote MATLAB software for simple filtering and peak detection. We removed high frequency noise from the SPG and PPG signals using a 6 Hz low pass filter and the local DC components by subtracting the values from a 500-point (2 second) moving average^38^. Next, a third order, 11-point Savitzky-Golay filter was applied to the signals for smoothing without peak distortion^39^. We wrote peak detection software for the SPG signal to identify the first peak, located immediately after the peak of the first derivative, for consistency (Fig. 7). Since the PPG signal was generally noisier than the SPG signal, and both signals were acquired from the same device, the PPG peak was located by finding the first peak after the SPG peak (Fig. 7). We calculated the intervals between peaks and saved them as text files for further processing. For completeness, we compared results from the peak of the first derivative for both signals (Supplementary Figs S1–S4) and the foot of both signals (Supplementary Figs S5–S8), defined as the trough immediately before the peak of the first derivative. The results for these two processing methods were less accurate for the SPG-based estimation of HRV.

We processed the text files of 5 minute intervals in Kubios with the default settings^23^. We manually corrected artifacts from the series of R-R intervals individually via the built-in thresholding function, without consulting the other available signals (e.g. the seated SPG signal was analyzed independent of the seated ECG signal for the same subject), to account for missed beats and poor peak detection. Artifact correction involved replacing the inaccurate R-R interval with a new interpolated interval based on the local surrounding intervals^23^. For calculation of the frequency domain results such as LF and HF, we applied the autoregressive approach, which has improved stability for shorter time series^40^.

### Statistical analysis

We plotted the Kubios HRV results from ECG, SPG, and PPG and then plotted the lines of best fit. We calculated the pairwise linear correlation coefficient for SPG versus ECG and PPG versus ECG. In addition, we generated corresponding Bland-Altman plots with 95% confidence limits of agreement^41^. We decided *a priori* that acceptable limits of agreement for HRV indices from SPG and PPG when compared to ECG would be within 20% variation of the mean ECG measurement^11^.

## Supplementary information

Supplemental information

## Data Availability

The datasets generated during and/or analyzed during the current study are available from the corresponding author upon reasonable request.

## References

[1] Thayer JF, Lane RD (2007). The role of vagal function in the risk for cardiovascular disease and mortality. Biol. Psychol..

[2] Thayer JF, Åhs F, Fredrikson M, Sollers JJ, Wager TD (2012). A meta-analysis of heart rate variability and neuroimaging studies: Implications for heart rate variability as a marker of stress and health. Neurosci. Biobehav. Rev..

[3] Dishman RK, Pattion RW, Smith J, Weinberg R, Jackson A (1987). Using perceived exertion to prescribe and monitor exercise training heart rate. Int. J. Sports Med..

[4] DONG JIN-GUO (2016). The role of heart rate variability in sports physiology. Experimental and Therapeutic Medicine.

[5] Flatt A (2018). Heart rate variability and training load among National Collegiate Athletic Association Division 1 college football players throughout spring samp. J. Strength Cond. Res..

[6] Senthinathan A, Mainwaring LM, Hutchison M (2017). Heart rate variability of athletes across concussion recovery milestones: A preliminary study. Clin. J. Sport. Med..

[7] Goessl VC, Curtiss JE, Hofmann SG (2017). The effect of heart rate variability biofeedback training on stress and anxiety: A meta-analysis. Psychol. Med..

[8] Gil E (2010). Photoplethysmography pulse rate variability as a surrogate measurement of heart rate variability during non-stationary conditions. Physiol. Meas..

[9] Parak, J. *et al*. Evaluation of the beat-to-beat detection accuracy of PulseOn Wearable Optical Heart Rate Monitor. *2015 37th Annu. Int. Conf. IEEE Eng. Med. Biol. Soc*. 8099–8102 (2015). 10.1109/EMBC.2015.7320273 26738173

[10] Lu S (2008). Can photoplethysmography variability serve as an alternative approach to obtain heart rate variability information?. J. Clin. Monit. Comput..

[11] Charlot K, Cornolo J, Brugniaux JV, Richalet JP, Pichon A (2009). Interchangeability between heart rate and photoplethysmography variabilities during sympathetic stimulations. Physiol. Meas..

[12] Schäfer A, Vagedes J (2013). How accurate is pulse rate variability as an estimate of heart rate variability? A review on studies comparing photoplethysmographic technology with an electrocardiogram. Int. J. Cardiol..

[13] Malik M (1996). Heart rate variability: Standards of measurement, physiological interpretation, and clinical use. Eur. Heart J..

[14] Plews, D. J. *et al*. Comparison of heart rate variability recording with smart phone photoplethysmographic, Polar H7 Chest Strap and electrocardiogram methods. *Int. J. Sports Physiol. Perform*. 1–17 (2017). 10.1123/ijspp.2016-0668 28290720

[15] Peng, R.-C., Zhou, X.-L., Lin, W.-H. & Zhang, Y.-T. Extraction of heart rate variability from smartphone photoplethysmograms. *Comput. Math. Methods Med*. 1–11 (2015). 10.1155/2015/516826 PMC4309304 25685174

[16] Kranjec J (2017). Design and clinical evaluation of a non-contact heart rate variability measuring device. Sensors.

[17] Boas DA, Dunn AK (2010). Laser speckle contrast imaging in biomedical optics. J. Biomed. Opt..

[18] Dunn CE, Lertsakdadet B, Crouzet C, Bahani A, Choi B (2018). Comparison of speckleplethysmographic (SPG) and photoplethysmographic (PPG) imaging by Monte Carlo simulations and *in vivo* measurements. Biomed. Opt. Express.

[19] Ghijsen M, Rice TB, Yang B, White SM, Tromberg BJ (2018). Wearable speckle plethysmography (SPG) for characterizing microvascular flow and resistance. Biomed. Opt. Express.

[20] Richards LM, Kazmi SMS, Davis JL, Olin KE, Dunn AK (2013). Low-cost laser speckle contrast imaging of blood flow using a webcam. Biomed. Opt. Express.

[21] Elgendi M (2012). On the analysis of fingertip photoplethysmogram signals. Curr. Cardiol. Rev..

[22] Feiner JR, Severinghaus JW, Bickler PE (2007). Dark skin decreases the accuracy of pulse oximeters at low oxygen saturation: The effects of oximeter probe type and gender. Anesth. Analg..

[23] Tarvainen MP, Niskanen J, Lipponen JA, Ranta-aho PO, Karjalainen PA (2013). Kubios HRV – Heart rate variability analysis software. Comput. Methods Programs Biomed..

[24] Laborde S, Mosley E, Thayer JF (2017). Heart rate variability and cardiac vagal tone in psychophysiological research – recommendations for experiment planning, data analysis, and data reporting. Front. Psychol..

[25] Nunan D, Sandercock GRH, Brodie DA (2010). A quantitative systematic review of normal values for short-term heart rate variability in healthy adults. PACE.

[26] Zhang Z (2015). Photoplethysmography-based heart rate monitoring in physical activities via joint sparse spectrum reconstruction. IEEE Trans. Biomed. Eng..

[27] Hayes MJ, Smith PR (2008). Artifact reduction in photoplethysmography. Appl. Opt..

[28] Sagaidachnyi AA, Skripal AV, Fomin A, Usanov D (2014). Determination of the amplitude and phase relationships between oscillations in skin temperature and photoplethysmography-measured blood flow in fingertips. Physiol. Meas..

[29] Shin HS, Lee C, Lee M (2009). Adaptive threshold method for the peak detection of photoplethysmographic waveform. Comput. Biol. Med..

[30] Lee B (2010). Improved elimination of motion artifacts from a photoplethysmographic signal using a Kalman smoother with simultaneous accelerometry. Physiol. Meas..

[31] Han, H., Kim, M. J. & Kim, J. Development of real-time motion artifact reduction algorithm for a wearable photoplethysmography. *Annu. Int. Conf. IEEE Eng. Med. Biol. - Proc*. 1538–1541 (2007). 10.1109/IEMBS.2007.4352596 18002262

[32] Brumfield AM, Andrew ME (2005). Digital pulse contour analysis: Investigating age-dependent indices of arterial compliance. Physiol. Meas..

[33] Abaji JP, Curnier D, Moore RD (2016). Persisting effects of concussion on heart rate variability during physical exertion. J. Neurotrama.

[34] Pertab JL, Merkley TL, Cramond AJ, Cramond K, Paxton H (2018). Concussion and the autonomic nervous system: An introduction to the field and the results of a systematic review. NeuroRehabilitation.

[35] Jianchu Y, Warren S (2004). A novel algorithm to separate motion artifacts from photoplethysmographic signals obtained with a reflectance pulse oximeter. 26th Annu. Int. Conf. IEEE Eng. Med. Biol. Soc..

[36] Choi B, Ramirez-San-Juan JC, Lotfi J, Nelson JS (2006). Linear response range characterization and *in vivo* application of laser speckle imaging of blood flow dynamics. J. Biomed. Opt..

[37] Ramirez-San-Juan JC, Ramos-García R, Guizar-Iturbide I, Martínez-Niconoff G, Choi B (2008). Impact of velocity distribution assumption on simplified laser speckle imaging equation. Opt. Express.

[38] Garde A, Karlen W, Dehkordi P, Ansermino JM, Dumont GA (2013). Empirical mode decomposition for respiratory and heart rate estimation from the photoplethysmogram. Comput. Cardiol. (2010)..

[39] Savitzky A, Golay MJE (1964). Smoothing and differentiation of data by simplified least squares procedures. Anal. Chem..

[40] Malliani A, Lombardi F, Pagani M (1994). Power spectrum analysis of heart rate variability: a tool to explore neural regulatory mechanisms. Br. Heart J..

[41] Giavarina D (2015). Lessons in biostatistics: Understanding Bland Altman analysis. Biochem. Medica.

