# Supplementary material for: Speckleplethysmographic (SPG) Estimation of Heart Rate Variability During an Orthostatic Challenge
Source: Sci Rep. 2019 Oct 1;9:14079. doi: 10.1038/s41598-019-50526-0 (PMC6773734; doi:10.1038/s41598-019-50526-0)
Supplement: Supplementary file 1 — Supplemental information [file 41598_2019_50526_MOESM1_ESM.docx]

**Speckleplethysmographic (SPG) Estimation of Heart Rate Variability During an Orthostatic Challenge**

Cody E. Dunn^1,2,3^, Derek C. Monroe^4^, Christian Crouzet^1,2^, James W. Hicks^5^, & Bernard Choi*^1,2,3,6^

^1^Beckman Laser Institute and Medical Clinic, University of California, Irvine, California 92612. ^2^Department of Biomedical Engineering, University of California, Irvine, California 92697. ^3^Edwards Lifesciences Center for Advanced Cardiovascular Technology, University of California, Irvine, California 92697. ^4^Department of Neurology, University of California, Irvine, California 92697. ^5^Department of Ecology and Evolutionary Biology, University of California, Irvine, California 92697. ^6^Department of Surgery, University of California, Irvine, California 92697. Correspondence should be addressed to BC (email: choib@uci.edu). *Corresponding author.

Supplementary Figures S1-S4 are the results of the analysis based on the peak of the first derivative of the SPG and PPG signals.

*S1. A box plot comparison of the percentage of artifacts corrected for ECG (green), SPG (blue), and PPG (black) during the orthostatic challenge (n=17). The circles represent outliers.*

*
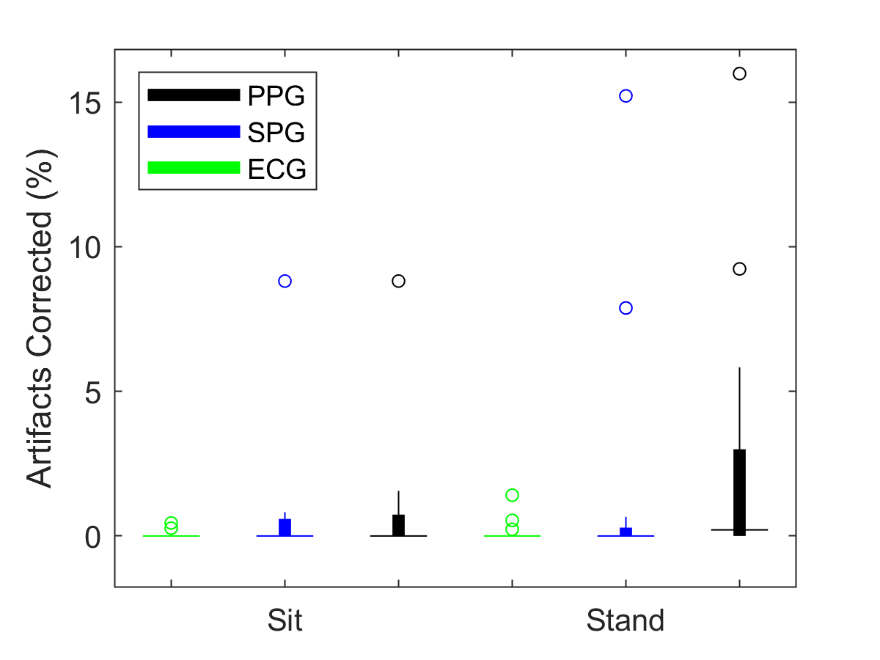
*

*S2. A comparison of SPG, PPG, and ECG HRV time domain parameters (SDNN; ms and RMSSD; ms) for the 17 subjects during both sitting and standing conditions. SPG and PPG are compared to ECG on scatter plots with a line of best fit and the line of best fit equation, the Pearson’s correlation coefficient, and significance in the upper left corner of the plot. Each Bland-Altman plot corresponds to the scatter plot directly above it, with the mean difference (black line), the 95% upper and lower limits of agreement (blue dashed lines), and the acceptable upper and lower limits of agreement (red dashed lines) also plotted. Please note that the Bland-Altman plots for SPG and PPG have different y-axis scales.*

*
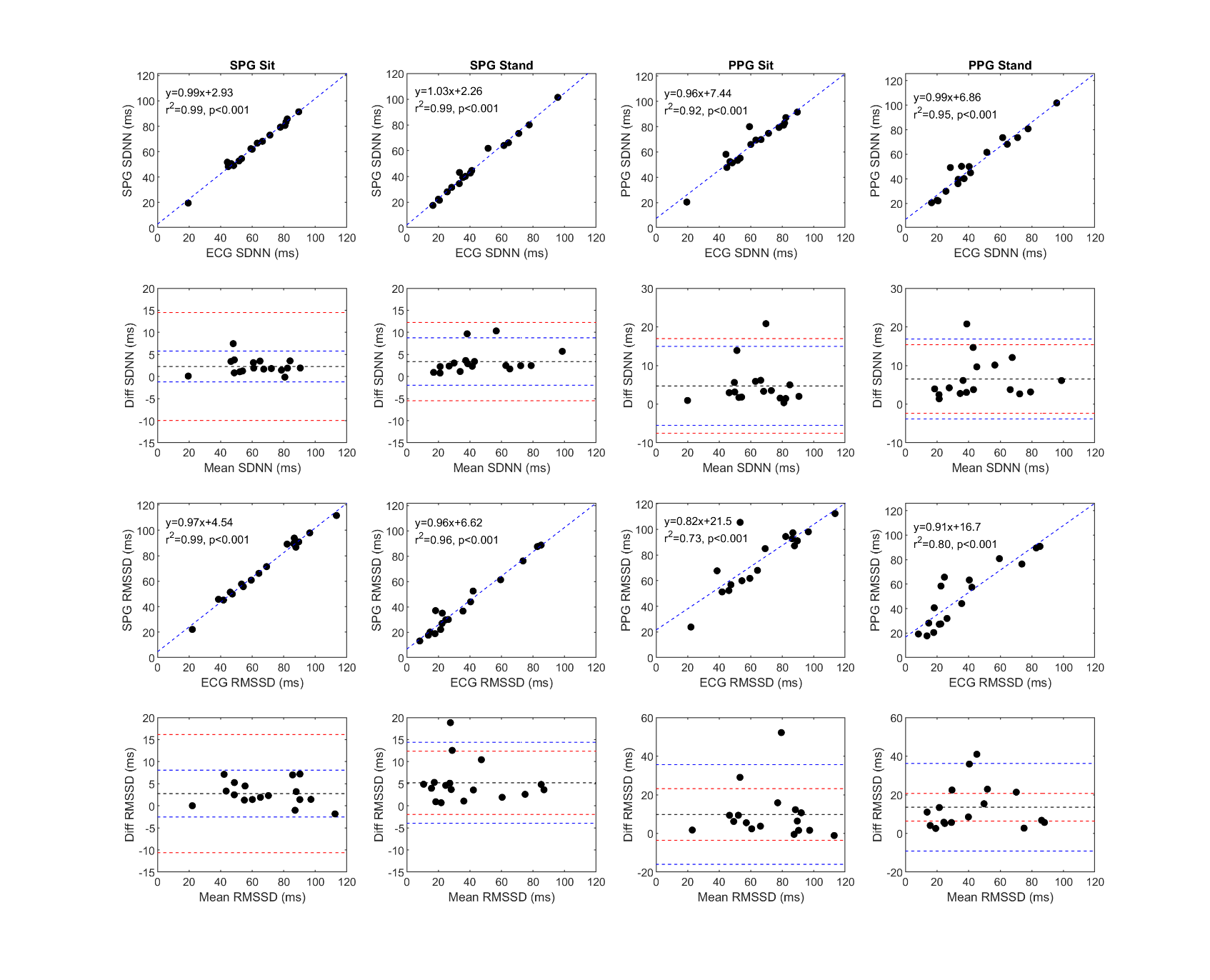
*

*S3. A comparison of SPG, PPG, and ECG HRV frequency domain parameters (LF; ms^2^ and HF; ms^2^) for the 17 subjects during both sitting and standing conditions. SPG and PPG are compared to ECG on scatter plots with a line of best fit and the line of best fit equation, the Pearson’s correlation coefficient, and significance in the upper left corner of the plot. Each Bland-Altman plot corresponds to the scatter plot directly above it, with the mean difference (black line), the 95% upper and lower limits of agreement (blue dashed lines), and the acceptable upper and lower limits of agreement (red dashed lines) also plotted. Please note that some of the Bland-Altman plots for SPG and PPG have different y-axis scales. The SPG LF correlation coefficient while seated based on first derivative peak detection is less than the same correlation coefficient based on standard peak detection (0.88 versus 0.99).*

*
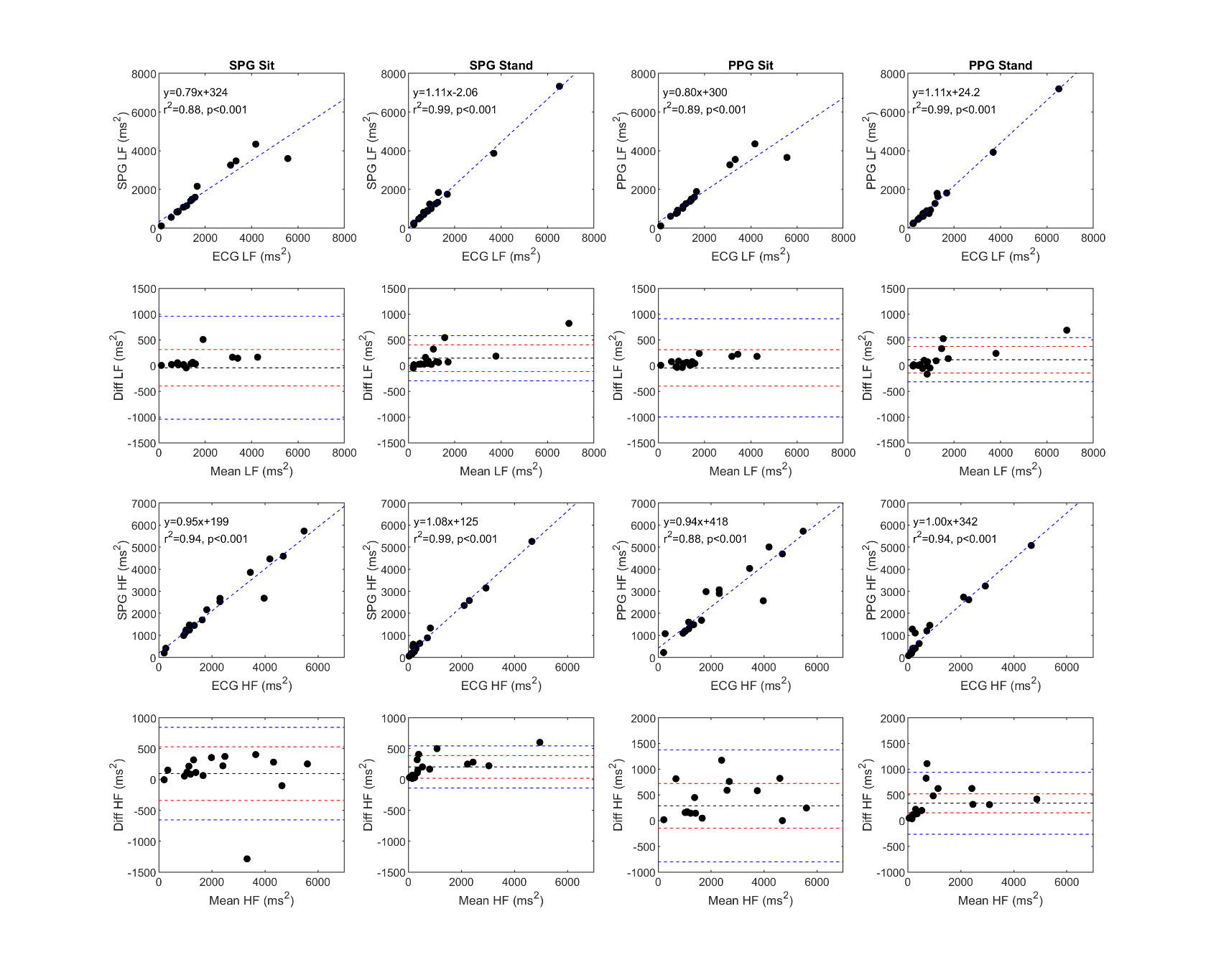
*

*S4. A comparison of additional SPG, PPG, and ECG HRV frequency domain parameters (normalized HF; normalized units (n.u.) and LF/HF; ratio) for the 17 subjects during both sitting and standing conditions. SPG and PPG are compared to ECG on scatter plots with a line of best fit and the line of best fit equation, the Pearson’s correlation coefficient, and significance in the upper left corner of the plot. Each Bland-Altman plot corresponds to the scatter plot directly above it, with the mean difference (black line), the 95% upper and lower limits of agreement (blue dashed lines), and the acceptable upper and lower limits of agreement (red dashed lines) also plotted.*

*
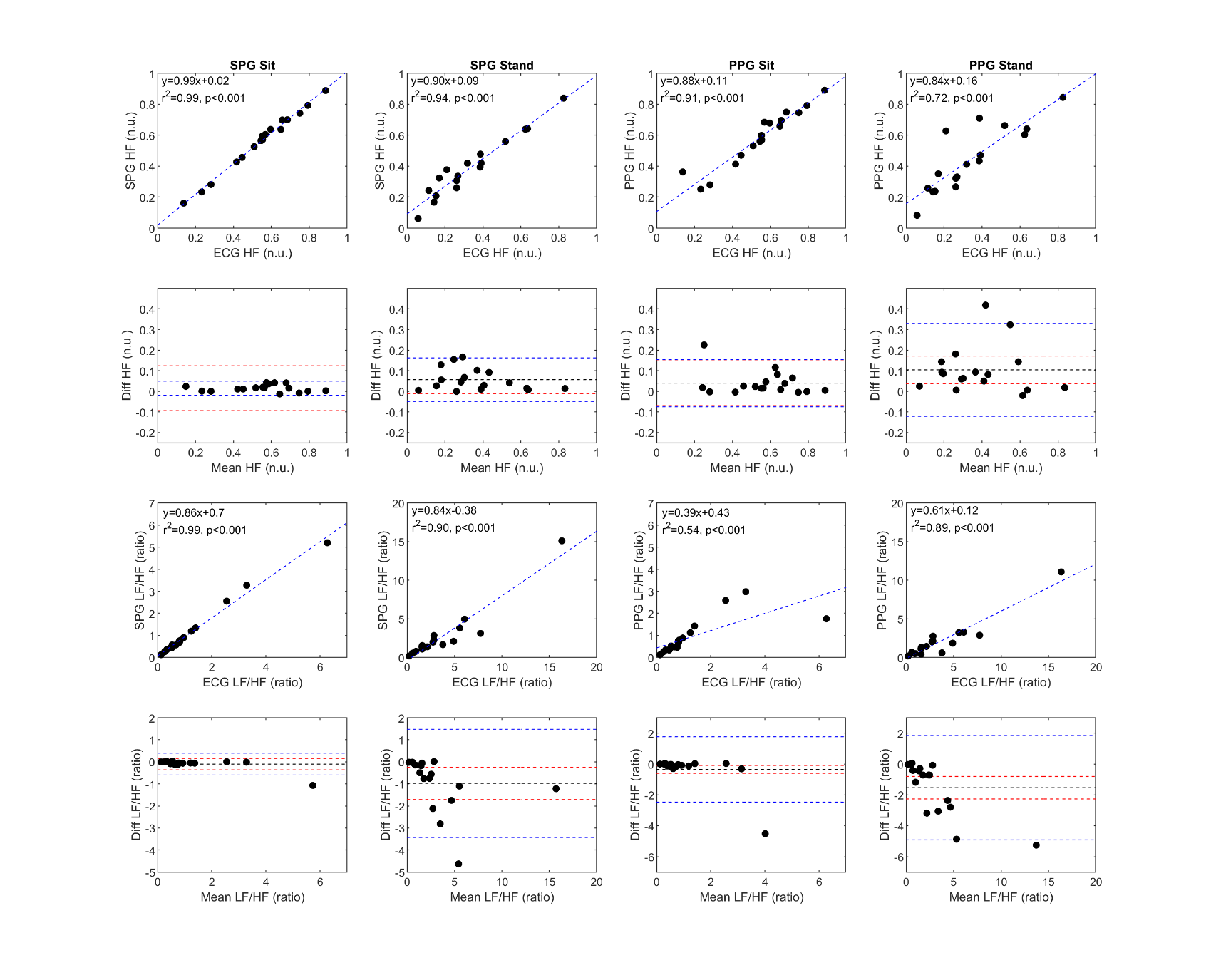
*

Supplementary Figures S5-S8 are the results of the analysis based on the foot that occurs immediately before the peak of the first derivative of the SPG and PPG signals.

*S5. A box plot comparison of the percentage of artifacts corrected for ECG (green), SPG (blue), and PPG (black) during the orthostatic challenge (n=17). The circles represent outliers.*

*
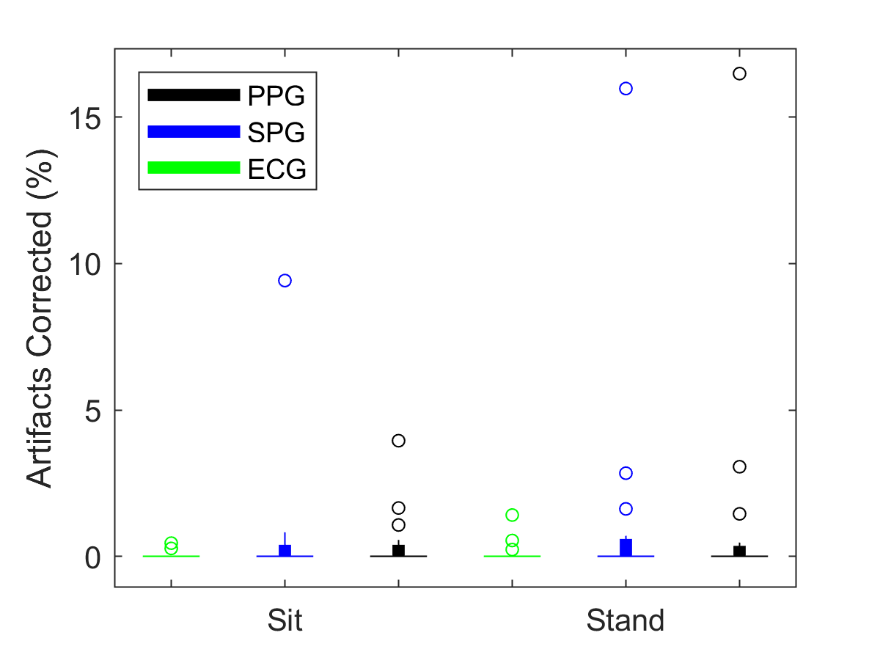
*

*S6. A comparison of SPG, PPG, and ECG HRV time domain parameters (SDNN; ms and RMSSD; ms) for the 17 subjects during both sitting and standing conditions. SPG and PPG are compared to ECG on scatter plots with a line of best fit and the line of best fit equation, the Pearson’s correlation coefficient, and significance in the upper left corner of the plot. Each Bland-Altman plot corresponds to the scatter plot directly above it, with the mean difference (black line), the 95% upper and lower limits of agreement (blue dashed lines), and the acceptable upper and lower limits of agreement (red dashed lines) also plotted. Please note that the Bland-Altman plots for SPG and PPG have different y-axis scales.*

*
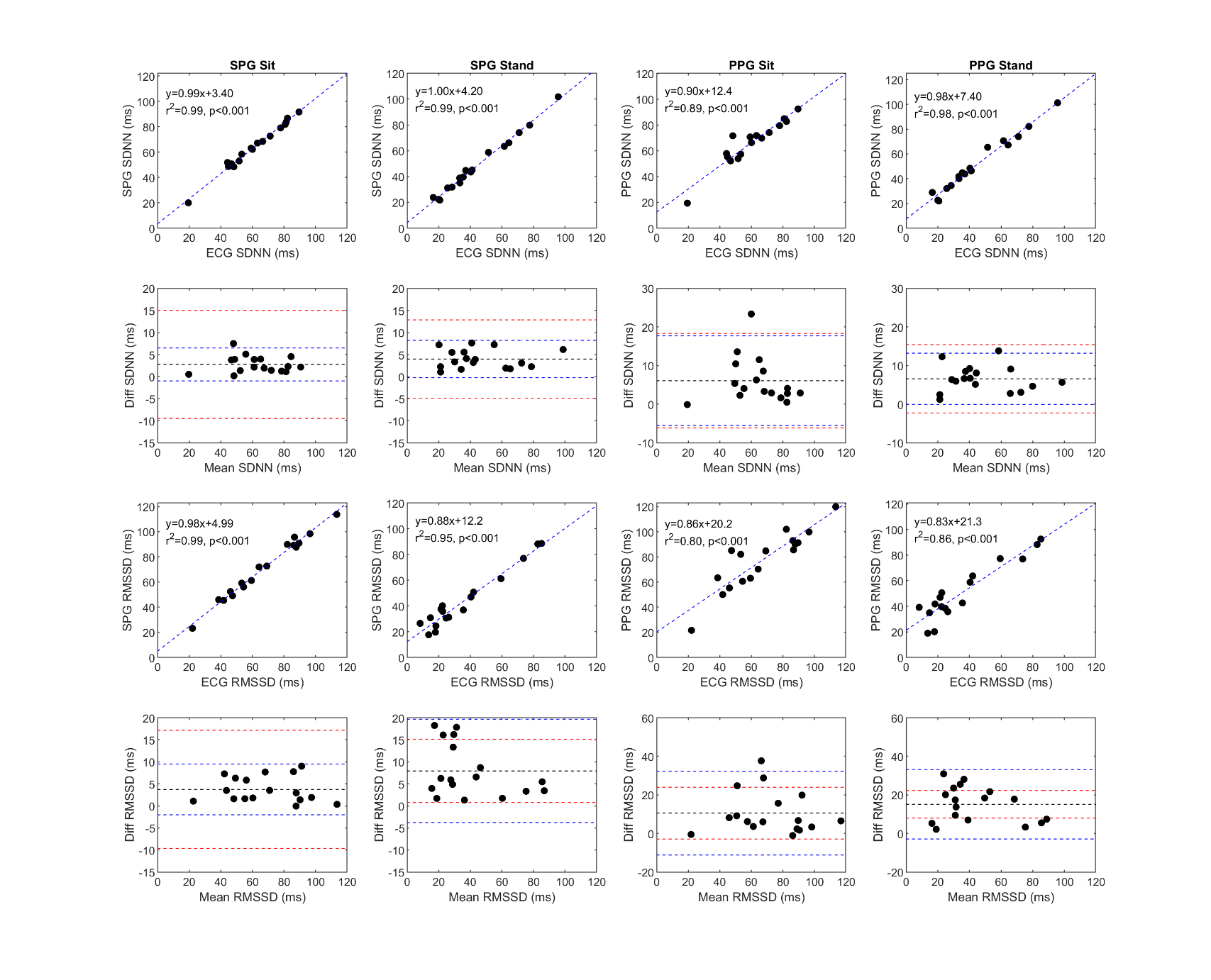
*

*S7. A comparison of SPG, PPG, and ECG HRV frequency domain parameters (LF; ms^2^ and HF; ms^2^) for the 17 subjects during both sitting and standing conditions. SPG and PPG are compared to ECG on scatter plots with a line of best fit and the line of best fit equation, the Pearson’s correlation coefficient, and significance in the upper left corner of the plot. Each Bland-Altman plot corresponds to the scatter plot directly above it, with the mean difference (black line), the 95% upper and lower limits of agreement (blue dashed lines), and the acceptable upper and lower limits of agreement (red dashed lines) also plotted. Please note that some of the Bland-Altman plots for SPG and PPG have different y-axis scales. The SPG LF correlation coefficient while seated based on foot detection is less than the same correlation coefficient based on peak detection (0.89 versus 0.99).*

*
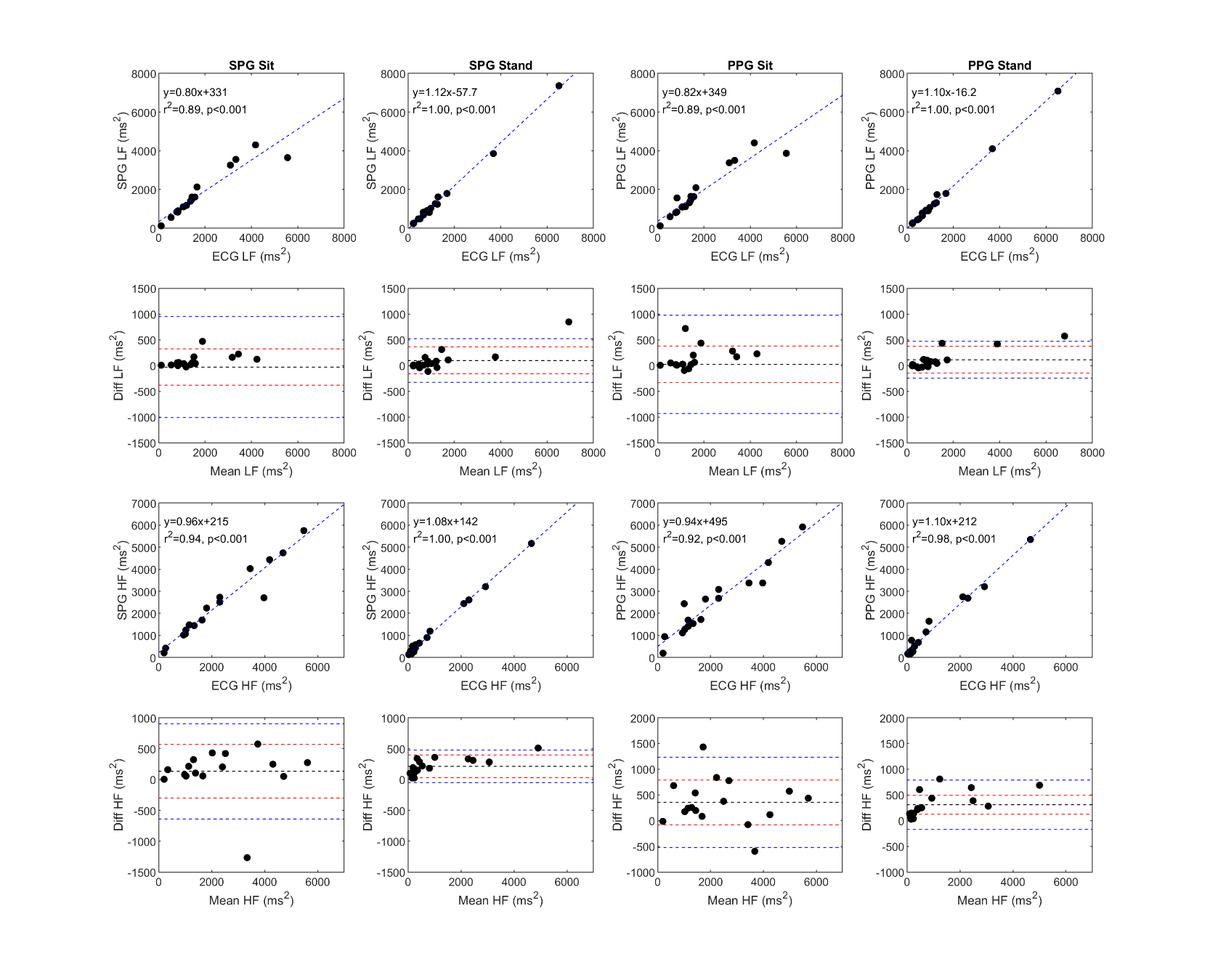
*

*S8. A comparison of additional SPG, PPG, and ECG HRV frequency domain parameters (normalized HF; normalized units (n.u.) and LF/HF; ratio) for the 17 subjects during both sitting and standing conditions. SPG and PPG are compared to ECG on scatter plots with a line of best fit and the line of best fit equation, the Pearson’s correlation coefficient, and significance in the upper left corner of the plot. Each Bland-Altman plot corresponds to the scatter plot directly above it, with the mean difference (black line), the 95% upper and lower limits of agreement (blue dashed lines), and the acceptable upper and lower limits of agreement (red dashed lines) also plotted. The SPG LF/HF correlation coefficient while standing based on foot detection is less than the same correlation coefficient based on peak detection (0.82 versus 0.94).*

*
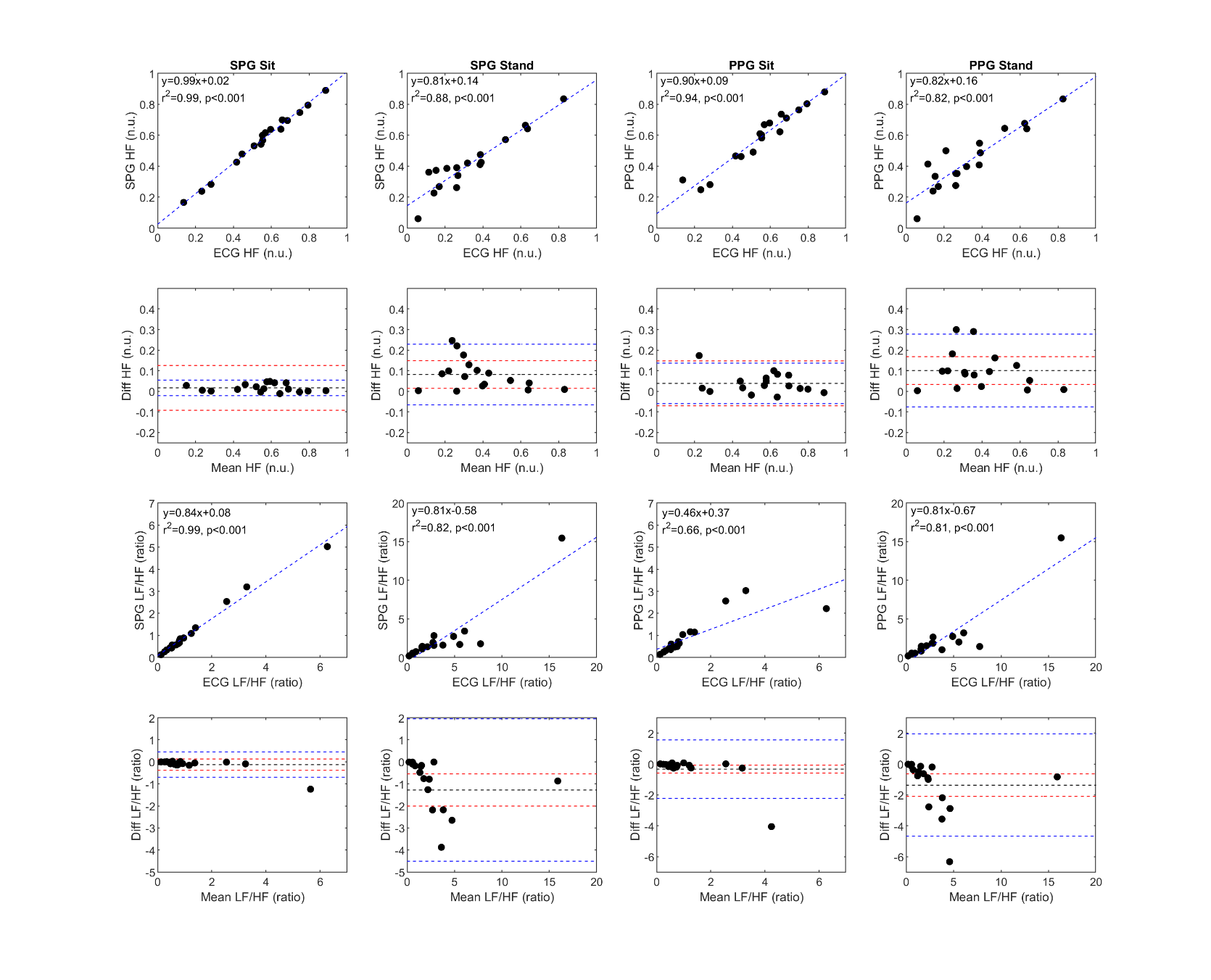
*
